# Water vapour and heat combine to elicit biting and biting persistence in tsetse

**DOI:** 10.1186/1756-3305-6-240

**Published:** 2013-08-19

**Authors:** Charles JF Chappuis, Steve Béguin, Michèle Vlimant, Patrick M Guerin

**Affiliations:** 1 Institute of Biology, Laboratory of Animal Physiology, University of Neuchâtel, Rue Emile-Argand 11, 2000, Neuchâtel, Switzerland

**Keywords:** Tsetse, *Glossina pallidipes*, Biting behaviour, Biting fly, Blood-feeding, Hygroreception, Thermoreception, Dry cell

## Abstract

**Background:**

Tsetse flies are obligatory blood feeders, accessing capillaries by piercing the skin of their hosts with the haustellum to suck blood. However, this behaviour presents a considerable risk as landing flies are exposed to predators as well as the host’s own defense reactions such as tail flicking. Achieving a successful blood meal within the shortest time span is therefore at a premium in tsetse, so feeding until replete normally lasts less than a minute. Biting in blood sucking insects is a multi-sensory response involving a range of physical and chemical stimuli. Here we investigated the role of heat and humidity emitted from host skin on the biting responses of *Glossina pallidipes,* which to our knowledge has not been fully studied in tsetse before.

**Methods:**

The onset and duration of the biting response of *G. pallidipes* was recorded by filming movements of its haustellum in response to rapid increases in temperature and/or relative humidity (RH) following exposure of the fly to two airflows. The electrophysiological responses of hygroreceptor cells in wall-pore sensilla on the palps of *G. pallidipes* to drops in RH were recorded using tungsten electrodes and the ultra-structure of these sensory cells was studied by scanning and transmission electron microscopy.

**Results:**

Both latency and proportion of tsetse biting are closely correlated to RH when accompanied by an increase of 13.1°C above ambient temperature but not for an increase of just 0.2°C. Biting persistence, as measured by the number of bites and the time spent biting, also increases with increasing RH accompanied by a 13.1°C increase in air temperature. Neurones in wall-pore sensilla on the palps respond to shifts in RH.

**Conclusions:**

Our results show that temperature acts synergistically with humidity to increase the rapidity and frequency of the biting response in tsetse above the levels induced by increasing temperature or humidity separately. Palp sensilla housing hygroreceptor cells, described here for the first time in tsetse, are involved in the perception of differences in RH.

## Background

Tsetse flies are obligate haematophagous insects that pierce the skin of the host with the haustellum to lacerate capillaries and suck blood. Tsetse are diurnal and their host-seeking behaviour is closely correlated to the activity of their hosts and predators [1]. Biting behaviour is the last step in host-seeking behaviour and represents a critical risk for tsetse. Indeed, upon landing on the host flies are exposed to predators and to the host’s defence such as grooming and tail flicking [2]. Tsetse are k-strategists and are among the dipterans with the longest life span [3], that can extend up to several months in the field [4]. Tsetse such as *Glossina pallidipes* feed every three to four days [5,6]. The trade-off between feeding and risk avoidance could explain the flies’ strategy of infrequent host visits but feeding to repletion when they do [2]. As blood is not directly available to tsetse on landing, cues emanating from the skin stimulate tsetse to bite where they can readily find blood, thereby minimising the time spent on the host and so reduce risk. The heat of mammalian skin elicits biting in tsetse [7] and in other ectoparasite arthropods [8-13]. But heat is not the unique factor in eliciting biting as it is a multi-modal sensory response, *i.e.* diverse stimuli can affect this behaviour. Van Naters*, et al.*[14] showed that the combination of a chemical cue, such as uric acid, and heat increased the time spent probing by *G. fuscipes fuscipes*. Gatehouse [15] also found that the probing response of the stable fly *Stomoxys calcitrans* was multi-modal as an increase in humidity with the addition of ammonia induced more flies to probe than increases in humidity alone. Later, Gatehouse [16,17] illustrated the importance of hygroreception in the biting responses of this species by showing that increases in humidity were as efficient at inducing the biting response as heat alone or the combination of humidity plus heat. The importance of humidity in host-seeking behaviour in tsetse was underlined by experiments conducted in a wind tunnel by Evans and Gooding [18]. They showed that an increase in both moisture and air temperature is better at eliciting upwind flight to a source of CO_2_ in *G. morsitans morsitans* than heated air alone. *Aedes aegypti* is also more attracted to combined heat and humidity increases than to heat alone in an olfactometer [19]. Heat and moisture emanating from the host [20,21] are clearly important host cues for haematophageous insects.

To our knowledge, the interaction of heat and humidity has not been investigated in the biting behaviour of tsetse. To study this we built an experimental setup that allows us to quantify the biting response of *G. pallidipes* to an increase in temperature accompanied by increasing humidity or to increasing humidity accompanied by a temperature increase of a fraction of a degree. We demonstrate how combined temperature and humidity increases serve to influence the biting response, response latency, biting persistence and the dynamics of this fundamental behaviour in tsetse. In addition, we report on neurones with hygroreceptive properties present in basiconic sensilla on the maxillary palps of *G. pallidipes.*

## Methods

### Insects

*G. pallidipes* (Austen) pupae were supplied by the International Atomic Energy Agency (IAEA) Siebersdorf Laboratories, Austria. The *G. pallidipes* colony originated from flies collected near Tororo, Uganda in 1975 that were reared successively at the University of Amsterdam, NL, the University of Bristol, UK, and at the IAEA-Siebersdorf Laboratories since 1986. Imagos were kept in 2 climate chambers offset by 2 hours with 10 h light at 26°C, 85% relative humidity (RH) and 14 h dark at 22°C, 85% RH. Sexes were separated at emergence. The behaviour of 3-day post-emergence unfed flies was observed during their two daily activity peaks in the first 1 h 30 min and last 2 hours of the photophase. The response of each fly was only tested once.

### Experimental set-up to measure tsetse biting

**Figure 1:**
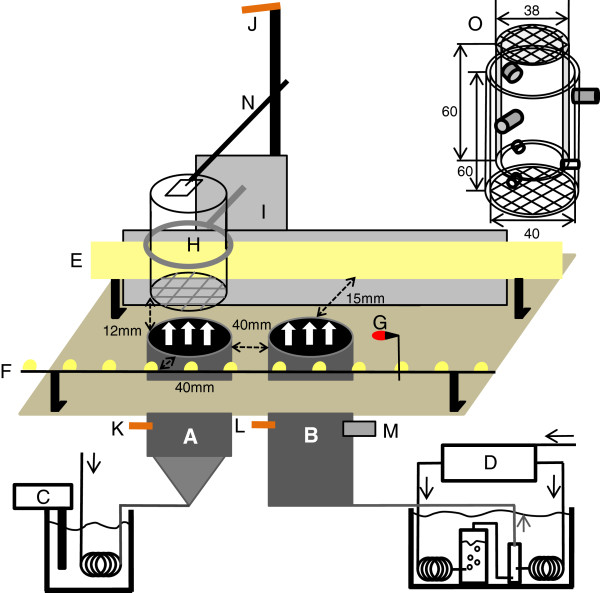
**Experimental setup used to record the biting responses of *G. pallidipes *(not to scale). A** acclimatisation airflow (the grey triangle symbolizes the funnel), **B** test airflow, **C** water-bath to control the temperature of the acclimatisation airflow, **D** apparatus to control the relative humidity and the temperature of the test airflow, **E** chain of power LEDs covered with opaque Plexiglas as background light, **F** chain of power LEDs to illuminate the underside of the cage, **G** red LED switched on by contact when the cage is in place on the test flow, **H** suspended cage, **I** reflective paper (grey colour), **J**, **K**, **L** thermistor probes measuring the ambient, acclimatisation and test airflow temperatures, the hygrometer **M** measuring the relative humidity of the test airflow and the anemometer **N**, **O** drawing of the insect cage in mm with a nylon mesh at the top of the inner tube and mosquito netting at the bottom of the outer tube. **H**, **I**, **J** and **N** were mounted on a sliding mechanism that allowed movement of the insect cage from the acclimatisation airflow to the test airflow.

The biting responses of individual *G. pallidipes* to rapid changes in temperature and/or RH were measured by moving a fly between two vertical airflows in a cage mounted on the side of an index card drawer used as a sliding mechanism (Figure 1). Cages were made of 2 Plexiglas® cylinders that fitted one inside the other with one end covered with grey mosquito netting (polyethylene, 1 mm mesh, bottom) and a nylon mesh (800 μm, Sefar AG, Heiden, St-Gallen, Switzerland, top; Figure 1). The cage was suspended with the mosquito netting 12 mm above the airflow (Figure 1). All flies were first exposed to an acclimatisation airflow that provided initial conditions at 24.5°C ± 0.1, 7.3% ± 0.8 RH (corresponding to a water partial pressure of 2.24 hPa), similar across all experiments (see below for measuring devices). This charcoal-filtered air flowed at 40-44 L/min through flow meters (Solartron Mobrey, Chanhassen, Minnesota, USA) and through two 11.5 m long copper coils (8 mm i.d.) immersed in a water-bath (LAUDA T, Lauda Dr. R. Wobser GmbH & Co, Lauda-Königshofen, Baden-Würtenberg, Germany; Figure 1). The copper coil outlets were joined in an insulated silicon tube to a 41 mm diameter glass funnel with a nylon screen (40 μm mesh, Sefar) to equalize air speed at its outlet (Figure 1). The funnel was mounted by a 35 mm i.d. aluminium tube (40 o.d., 65 mm long, Figure 1). The test airflow was produced by the apparatus described in Taneja and Guerin [22]. Briefly, charcoal-cleaned air passed through two copper coils immersed in a water-bath. One flow was humidified in a saturation vessel filled with distilled water. Using flow controllers, the two flows were mixed at a defined ratio in a chamber whose outlet was connected via a water-jacketed Teflon® tube to a stainless-steel water-jacketed tube (35 mm i.d., 255 mm long; Figure 1). The desired RH was obtained by adjusting the flow rates of the humidified and dry flows at an air speed of 0.7 m/s (Thermo-air anemometer, Schiltknecht Messtchnik, Gossau, Zürich, Switzerland). Airflow and ambient air temperatures were measured with three thermistor probes (Sable Systems, Las Vegas, Nevada, USA). RH of the test airflow was continuously recorded with a hygrometer (HMP50-60, Vaisala, Helsinki, Finland) and the same probe was used to measure that of the acclimatisation airflow each day before starting experiments. Thermistor probes and the hygrometer were connected to an interface (UI-2, Sable Systems) and signals were recorded with Expdata (version: 1.2.1, Sable Systems). An anemometer (Thermo-air anemometer, above) was placed 10-20 mm above the centre of the cage to record the passage of the test airflow through it (Figure 1). Ambient temperature and RH in the experimental room were measured at 22.3°C ± 0.7 and 48.3% ± 2.2 during experiments.

### Filming the biting response

The biting behaviour of *G. pallidipes* was recorded with two cameras (Full HD mode, Casio Exilim EX-ZR100, Shibuya, Tokyo, Japan) oriented to film the fly haustellum crossing the mosquito netting (Figure 1; Additional file 1). Background was provided by retroreflective paper (Scotchlite 680-CR, 3 M, Rüschlikon, Zürich, Switzerland; Figure 1) and by a string of LEDs (Power LED warm white, 30 spaced equidistant at 15 mm; Luniscontrol Gmbh, Lanzenhäusern, Bern, Switzerland) covered with an opaque sheet of Plexiglas® to equalize light intensity (Figure 1). Additional light was used to illuminate the lower part of the cage with a string of similarly mounted LEDs tilted at an angle of 45° on the same side of the cameras. The fly holding cage closed a contact that caused a red LED to light up (visible on the video) as a record of the arrival of the cage over the test airflow (Figure 1). Light intensity was 1440 lux at the cage lower screen.

Flies, in batches of 10, were placed individually in cages and held in the dark of the room where experiments were conducted. Timing was co-ordinated using a stopwatch. At time 0 s, recording of temperature, RH and wind speed was launched. At 5 s the first camera began filming and at 15 s the cage was moved into the acclimatisation airflow for 120 s. At 125 s, the second camera started to film and at 135 s the cage was moved into the test airflow to expose the fly for 120 s to the test conditions listed in Table 1.

**Table 1:** Temperature, RH and corresponding partial pressure of water vapour differences in the air streams

**Temperature increase [°C]**	**RH increase [%]**	**Partial pressure of water vapor increase [hPa]**	**Number of flies tested**		
			**Male**	**Female**	**Total**
0.2 ± 0.2	1.1 ± 0.5	0.4 ± 0.2	20	21	41
	10.8 ± 1.1	3.4 ± 0.3	19	20	39
	60.6 ± 0.4	18.0 ± 0.2	30	20	50
	75.4 ± 0.7	23.5 ± 0.5	20	20	40
3.0 ± 0.2	1.4 ± 0.04	1.0 ± 0.02	0	10	10
	11.1 ± 0.3	4.5 ± 0.1	0	10	10
	60.0 ± 0.4	22.4 ± 0.2	0	10	10
13.1 ± 0.1	0.2 ± 0.2	2.7 ± 0.2	14	20	34
	10.5 ± 0.4	9.4 ± 0.3	20	28	48
	34.5 ± 0.6	24.7 ± 0.3	20	20	40
	60.0 ± 0.6	41.8 ± 0.5	20	20	40
	72.4 ± 1.3	49.8 ± 0.8	21	18	39

Values are means ± SD for the physical parameters; number of flies of each sex tested with each treatment is also provided. Water vapour partial pressure was calculated for treatments by multiplying the RH by the saturation pressure [23] at an atmospheric pressure of 951 hPa.

### Analysis of the biting responses

To quantify biting, a bite was defined as the act of passing the haustellum through the netting (Additional file 1). No substrate was supplied beneath the mosquito netting, and flies were only exposed to the temperature and humidity stimuli provided in the air streams. The time the red LED switched on and each time the haustellum crossed the netting and was withdrawn, was recorded with the video analysis programme Kinovea (version: 0.8.14) [24]. For statistical analysis, the program R (version: 2.15.0) [25] was used and the level of significance was set at 0.05.

Proportions of responding flies were analyzed using a generalized linear model (GLM) with a logit link function. Latency was measured as the time between the moment the cage arrived in the test flow (red light on) and the onset of the first bite. To determine if a treatment increased or reduced latency, Cox proportional hazards regression was used with the R package *survival* to allow accounting for flies that did not respond as censored data.

As flies bit successively, the number of bites was log transformed and analysed by a factorial ANOVA, following a test for homogeneity of variance with a Bartlett test. Multi comparisons between treatments were made with Tukey’s Honest Significant Difference test (Tukey HSD). Data on time spent biting, average time per bite and average time between successive bites were analysed with a GLM with a reciprocal link function (Gamma family). Where necessary, multi comparison tests were done with a GLHT function depending on the model used (below) with a Tukey matrix of contrast (R-package: *multcomp*). As the number of responding flies exposed to an increase of 0.2% RH and 13.1°C was low (5 over 34 individuals tested), these data were pooled with data obtained for an increment of 10.5% RH at the same temperature increase. No significant difference in deviance was observed by pooling these two categories (ANOVA). Each step in reducing the Cox model and GLM was controlled by a deviance analysis following a *Χ*^2^ distribution (ANOVA). Temperature, RH, partial pressure and saturation pressure of water for experiments were analysed with Expdata (version: 1.2.1, Sable Systems) and are shown in Table 1.

### Electron microscopy

**Figure 2:**
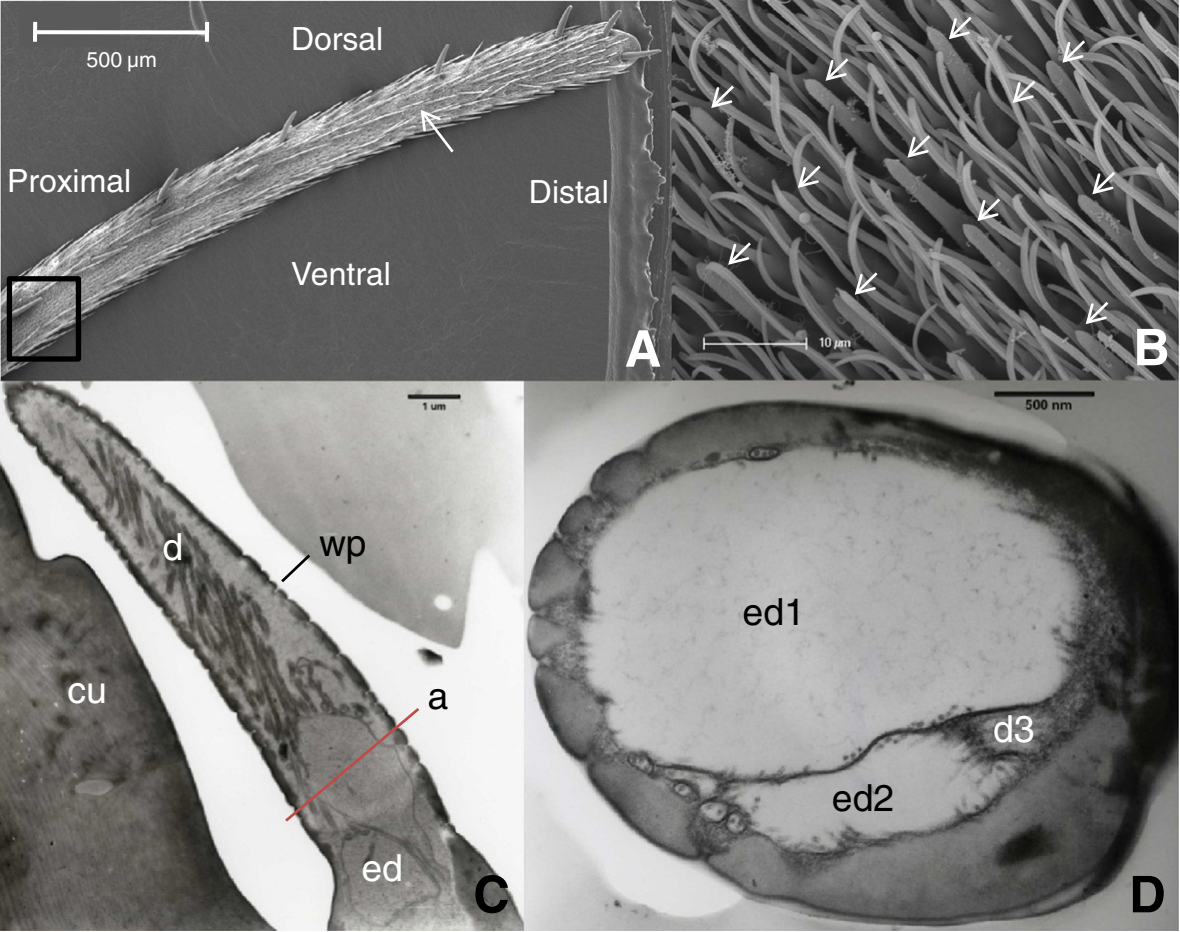
**Micrographs showing the location and the fine structure of basiconic sensilla on *G. pallidipes *palps. A** Image of the right palp with basoconic sensilla along the lateral groove delimited between the inside of the box proximally and the white arrow distally. **B** Magnified proximal zone (box in A) showing numerous basiconic sensilla (arrows) surrounded by microtrichae. **C** Longitudinal section of a sensillum with a thin porous wall (wp), ramified dendrites (d), two enlarged dendrites (ed) at the base and cuticle (cu). **D** Cross section at level a in C showing two enlarged dendrites (ed1 and ed2) and the third unbranched dendrite (d3). The scale bar is 500, 10, 1 μm and 500 nm for **A**, **B**, **C** and **D**, respectively.

Sample preparations for scanning and transmission electron microscopy were made following protocols described in Kessler*, et al.*[26]. For scanning electron microscopy, excised heads of *G. pallidipes* were fixed in 70% ethanol, dehydrated gradually in acetone and desiccated by critical point drying using CO_2_ (Bal-Tec CPD 030, Balzers, Liechtenstein). For transmission electron microscopy, whole heads of female and male *G. pallidipes* were immersed in Karnovsky fixative (pH 7.4) at a sucrose concentration of 4% and 10% to test if swelling of outer dendrites was not due to a hypotonic effect. The palps of 12 female and two male *G. pallidipes* were serially cut in cross and longitudinal sections. Thin sections (100 nm) were collected every 2 μm over 20 μm. For four females, thin sagittal sections of the basal parts of sensilla across the axis of the palp (from the ventral to the dorsal side) were examined over 4 μm along the lateral external palpal groove (Figure 2). To determine the arrangement of dendrites, eight basiconic sensilla of four flies were cut serially in 100 nm thick sections and every section was examined from half way along the sensillum to the ciliary roots.

### Electrophysiological recording from palp sensilla

A modified version of the set up used by Taneja and Guerin [27] was used to record action potentials from receptor cells within basiconic sensilla on the palps of *G. pallidipes*. After anesthesia with CO_2_, the fly was immobilized in a disposable micropipette tip (Kartell Spa, Noviglio, Lombardia, Italy) cut at the tip and held horizontally with the head protruding at the narrow end. The disengaged haustellum was attached to the tip with sticky tape. A V-shaped silver reference electrode was placed between the protruding head and the end of the micropipette tip. Mouthparts, except the maxillary palps, and eyes were embedded in dental cement (Ketac™ Cem radiopaque, 3 M ESPE, Seefeld, Bayern, Germany) to prevent movement of the head. A 0.1 mm diam. tungsten recording electrode sharpened by electro polishing in saturated KNO_2_ solution was placed randomly in the proximal 2 mm of the lateral external palp groove (Figure 2) and connected, in succession, to a high impedance preamplifier (×10; Syntech, Kirchzarten, Baden-Württemberg, Germany), an AC/DC amplifier (UN-03, Syntech) and the intelligent data acquisition controller (IDAC) analog-digital converter (USB-IDAC box, Syntech) to a computer. Spikes were recorded with Autospike (Syntech). Spikes were considered when the amplitude was greater than 1.9 - 2 times the standard deviation of the entire signal amplitude. The number of spikes counted for the first 2 s of stimulation was divided by 2 to estimate the spike frequency in Hz. Statistical analyses of spike frequencies were performed by ANOVA following a test for homogeneity of variance with a Bartlett test and multi comparisons between treatments were made with Tukey’s Honest Significant Difference test.

To test the responses of neurones to changes in RH, palps were exposed to five RH drops from 100% (−0.02% ± 0.3; -26.4% ± 1.7; -44.1% ± 4.0; -69.4% ± 4.8; -91.1% ± 3.4), obtained by mixing moist and dry air at different proportions with a stimulator. For this, two airflows were mixed by manually adjusting two flow controllers (C05K510418, Norgren, Lichfield, Staffordshire, United Kingdom) for a desired moist to dry air ratio. The flow controllers mounted on solenoid valves (RPE 3105 NC 230 V/AC, RPE S.r.l., 22070 Carbonate, Lombardia – CO – Como, Italy) were arranged in six pairs providing six channels triggered by an external electronic control that allowed the manual selection of the desired RH shift. Moist and dry airflows were produced by splitting charcoal-filtered air at 0.4 bar into two channels isolated from each other with non-return valves (T51P0008, Norgren). The moist channel was first humidified by bubbling it through a 2 L gas-wash bottle filled with distilled water immersed in a water-bath at 35°C. This humidified air was cooled to room temperature (25°C) by passing it through a copper coil before bubbling it through a second glass bottle (1 L) filled with distilled water immersed in a second water-bath at room temperature. The dry channel air passed through copper tubes immersed in the same water-baths as the moist airflow. Stimulation with RH drops was made by simultaneously closing the first water-saturated channel and opening one of the five other channels for 8 s. At the output, RH of the airflow was measured with a hygrometer (HMP50-60, above). The air speed at the output of the stimulator was measured with an anemometer (Thermo-air anemometer, above) and set at 1 m/s. Temperature of the output airflow was measured daily with a thermistor (PT-6 Physitemp, Science products GmbH, Hofheim, Hessen, Germany) at 25.4°C ± 0.2 for the water-saturated flow and 25.0°C ± 0.4 for the dry flow (8.9% RH).

## Results

### Biting responses to a temperature increment of 13.1°C and varying RH increments

**Figure 3:**
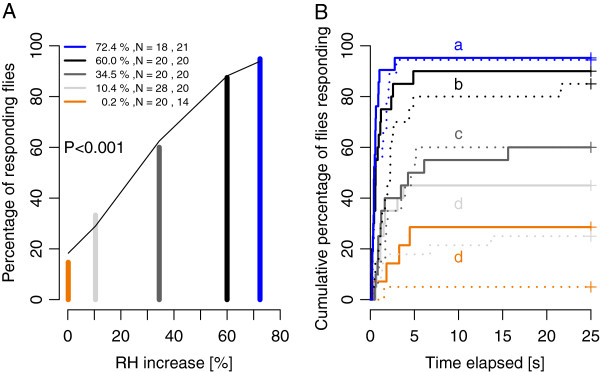
**Proportion of flies biting and response latency for increases in RH at a 13.1°C air temperature increase. A** Proportion of flies that bite at least once. Line shows the predicted values from the GLM model. **B** Proportion of flies biting as a function of the response latency. Recording time was 120 s but only the first 25 s is presented as latency did not exceed this. Curves with different letters are significantly different. Solid lines are for males and dotted lines are for females.

**Figure 4:**
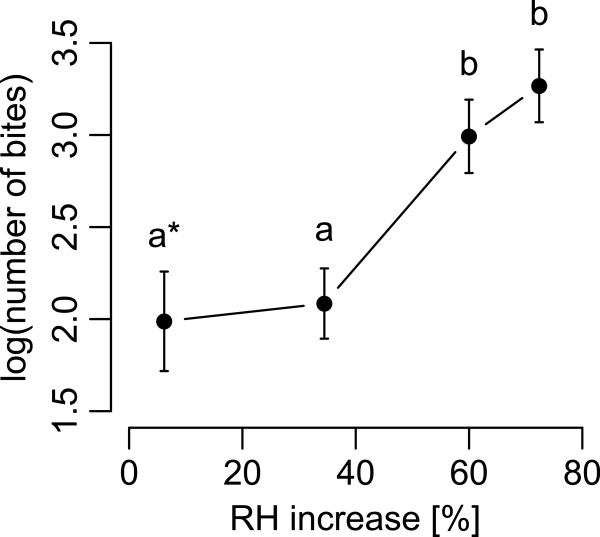
**Number of bites per fly at increasing RH increments with a 13.1°C air temperature increase.** Log transformed mean number of bites (± SEM) with different letters are significantly different following the Tukey HSD post-hoc test. As the number of flies responding to an increment in RH of 0.19% was very low (5 over 34 individuals tested) these data were pooled with responses obtained for an increment of 10.45% (asterisk).

An increase of 13.1°C brought the air stream temperature near that of the host. Under these conditions the proportion of flies that bit at least once increased significantly (GLM, P < 0.001) with increasing RH increments to reach 94.9% for an increment of 72.3% (Figure 3A). Latency of flies exposed to RH increments of 10.5%, 34.5%, 60% and 72.4% was, respectively, 2.6, 5.1, 12.8, 24.3 times shorter than flies exposed to the minimal RH increment of 0.2% (Figures 3B). The unique difference found between males and females was in latency as males responded 1.7 times faster than females (significant intercept of the Cox regression for the sex variable, P < 0.01). The number of bites increased significantly (ANOVA, F_3,113_ = 8.689, P < 0.001) with increasing RH increments (Figure 4). Increments above 34.5% RH significantly affected the number of bites but increments below 34.5% RH did not, *i.e.* flies exposed to the RH increase of 72.4% bit 26 times (detransformed mean), 3.3 times more than flies exposed to a RH increase of 34.5% (detransformed mean of 8 times, Figure 4).

**Figure 5:**
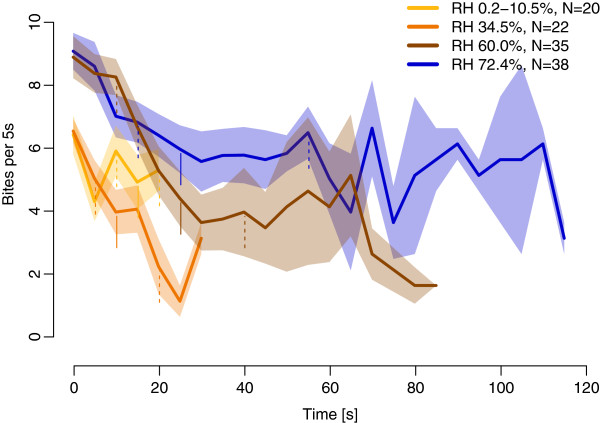
**Biting frequency dynamics over time for RH increments with a 13.1°C air temperature increase.** The frequency was calculated for each RH increment (different colours) as the number of bites per 5 s and presented as the average biting frequency (bold lines) ± SEM (shaded areas). Time 0 is the onset of the first bite. All flies did not bite for 120 s so medians (vertical solid lines) for the time spent biting by flies are drawn for each RH increment; vertical dashed lines to the left of the median represent the first quartile and to the right the third quartile.

The time spent biting was positively correlated with increasing RH (Additional file 2A). Only the highest RH increments significantly increased the biting time, compared to the lowest increase. The ratio between the time spent biting by flies exposed to RH increments of 72.4% and 34.5% is 2.1, lower than the ratio obtained for the number of bites for these two treatments (see above). This indicates a change in either average time between successive bites or in the average time of a bite between the two RH levels. In fact the average time per bite was significantly shorter for flies exposed to 72.4% than flies exposed to 34.5% RH (Additional file 2B). No significant difference was found between interbite intervals as a function of humidity (medians of 0.43 s, 0.39 s, 0.35 s, 0.4 s for RH increments of 6.2%, 34.5%, 60%, 72.4%, respectively; GLM, P = 0.67). The highest mean frequency of biting at 8.45 per 5 s was recorded for the highest RH increment (Figure 5; Additional file 1).

All parameters used above to describe the biting behaviour of *G. pallidipes* do not take into account the fact that the biting response occurs over time. It appears that flies did not bite with the regular frequency of a standing wave (Figure 5). In the first 5 seconds the frequency was at its maximum: 5.8, 5.9, 8.3, 8.4 bites per 5 s, respectively, at RH increments of 0.2-10.5%, 34.5%, 60% and 72.4% and then biting frequency decreased. This drop in biting frequency was related to RH and was highest for the 34.5% RH increase and lowest for the 72.3% RH increase. This resulted in biting termination at 30s for the 22 responding flies at the 34.5% RH increase, at 85 s for 35 responding flies at the 60% RH increase, and one fly was still biting at 120 s of the 38 responding flies at the 72.4% RH increase (Figure 5).

### Biting responses to temperature increments of 0.2°C and 3°C and varying RH increments

When the temperature shift was minimized to 0.2°C, RH increments of any size did not significantly (GLM, P = 0.092) increase the proportion of flies biting: of the tested flies (Table 1), 0, 0, 10 and 2.5% responded to the RH increments of 1.1, 10.8, 60.6, 75.4%, respectively. Furthermore, when females were exposed to an increase of 3°C accompanying RH increases of 1.4, 11 and 60% (Table 1) only one of 10 females responded to the 11% RH increase and the other RH increases failed to elicit biting.

### Biting responses to minimal RH increments

When RH increases were minimized to less than 1.5%, only a temperature increase of 13.1°C succeeded in eliciting biting: 14.7% of the 34 flies tested responded (Figure 3A). However, this comparison needs to be qualified: RH increases at different temperatures do not produce the same increase of water vapour pressure in air (Table 1). An increase of 13.1°C and 0.2% RH results in water vapour pressure of 2.7 hPa, roughly the same order as produced by an increase of 0.2°C with a 10.8% RH increase (3.4 hPa) or by a 3°C increase with a 11.1% RH increase (4.5 hPa). Nevertheless, with similar minimal water vapour increases of between 2.7 to 4.5 hPa none of the 39 flies responded to an increase of 0.2°C and only one female out of 10 responded to a 3°C increase.

Whereas RH increases accompanying minimal temperature increases or temperature increases accompanying minimal RH increases succeeded in inducing biting in no more than 14.7% of flies, the proportion of responding flies reached nearly 95% when both temperature and humidity were increased by 13.1°C and 72.4% RH (Figure 3A). This indicates a strong synergism between RH and temperature.

### Neuroanatomy of palp basiconic sensilla

On the lateral side of each palp at approximately 800 μm from the tip, a ventro-lateral field of wall-pore sensilla extends over a distance of 1200 μm and is 30 μm in width (Figure 2A & B). These hairs are wall-pore single-walled (wpsw) basiconic sensilla (Figure 2C). They are short (10–15 μm in length) with a diameter of 1.6 μm in the middle and 2.6 μm at the base (Figure 2C). The cuticular apparatus is a thin single wall (100 nm thick) pierced by numerous pores (30–40 nm in diameter) with pore-tubules extending into the lumen shaft. The lymphatic space contains few or more dendritic branches (up to 72) and can be filled with filaments which make it electron dense (Figure 2C).

At the base of the sensillum three sensory cells are associated with three enveloping cells (thecogen, tricogen and tormogen). From the thecogen cell a short sheath emerges surrounding the outer dendritic segments. To describe the fine structure of the outer dendritic segments and their branching pattern, all of the 56 sensilla examined (18 flies) presented two swollen dendritic segments at the base of the sensilla (Figure 2C & D). The swelling of the outer dendrites is not due to an uptake of water driven by a hypotonic fixing solution, as the same swelling was also found using a hypertonic fixative.

### Eletcrophysiology responses of dry cells

**Figure 6:**
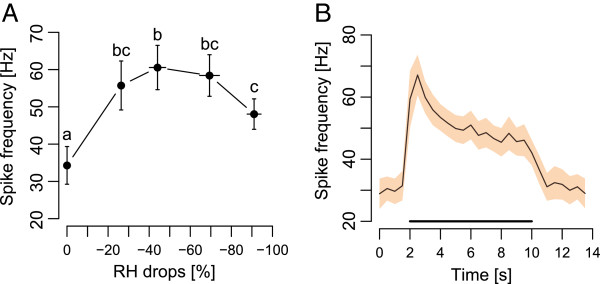
**Neuronal responses in *G. pallidipes *palp basiconic sensilla to RH decreases in the air. A** Mean neuronal responses (± SEM) recorded from 17 basiconic sensilla on 1 female and 4 male *G. pallidipes* to RH decreases (from 100%). Spike frequencies with different letters are significantly different. **B** Mean responses of neurones in the same 17 sensilla (± SEM, shaded area) to a RH decrease of −44.1% for 8 s (bold black bar). The spike frequency is the integration of the number of spike over 500 ms bins.

Neuronal responses to RH decreases were recorded from 17 wpsw basiconic sensilla of four male and one female *G. pallidipes*. In all of the 17 sensilla tested, the spike frequency of neurones increased by at least a factor of 1.5 when RH was decreased. Spike frequency increased significantly with RH drops (ANOVA, F_4,81_ = 12.86, P < 0.001; Figure 6A). The spike frequency reached a plateau between a drop of 44% and 69% RH and decreased significantly for a drop of 91% RH (Figure 6A). The response of neurones to RH drops of 44% showed a phasic-tonic response pattern (Figures 6B): the spike frequency increased from 30 Hz to 67 Hz in the first second of stimulation and then decreased to 45 Hz but neurones did not completely adapt to the 8 s RH drop (Figure 6B). After stimulation, when the RH of the airflow returned to the 100% RH level, the spike frequency decreased to the pre-stimulation level (Figure 6B). An example of the responses recorded from a sensillum housing a dry cell to RH drops of 0.1%, 27.4% and 41.4% is provided (Additional file 3). During preliminary experiments, only four sensilla of 60 tested presented a neurone that responded to an increase in RH.

## Discussion

Our results show that combined RH and temperature increases act synergistically to induce the biting response in tsetse. Moreover, at a temperature increment of 13.1°C, as RH increased, the flies responded more rapidly and in higher numbers. The persistence of biting behaviour measured by the number of bites and the time spent biting also increased with increasing RH. The dynamics of the biting response correlated over time with the amount of water in warm air, with a biting frequency that was higher at the beginning of the 2 min exposure and then fell off rapidly, but flies adapted more slowly at higher RH increments.

Latency and the proportion of flies responding are, respectively, negatively and positively correlated to RH increases at the 13.1°C air temperature increment. This indicates a very close link between the integration of thermoreception and hygroreception to initiate biting behaviour in tsetse. *G. pallidipes* and other savannah species exploit mainly mammals to obtain a blood meal [28,29]. These hosts lose heat and water through their skin [21], creating a humidity gradient between the skin and the environment [20,30,31], equipping these animals with a means of regulating body temperature through evaporation. Indeed, water droplets are formed by aprocrine glands during sweating [32] indicating that the humidity is close to saturation in the air near the skin (>90% at 37°C, 57 hPa). Taking into account the environmental conditions prevailing during the dry season in Africa (<40% at 30°C, 17 hPa) [1], differences in water partial pressure of more than 40 hPa could occur between a vertebrate host and its environment during the dry season and in the range of 38 hPa during the wet season (60% at 25°C, 19 hPa). This corresponds to the range of water vapour partial pressure differences tested here. The combination of heat and water vapour assist tsetse to find hosts as demonstrated by Evans and Gooding [18] and we show here how temperature and humidity combined serve to elicit the biting response at the feeding site. Our findings corroborate those of Khan and Maibach [33] who found that heat must be combined with humidity to elicit biting in another dipteran, *Ae. aegypti*. Previous studies have demonstrated that heat elicits a biting response in tsetse flies as described by Dethier [7] and later by Brady [34] and this has been amply exploited in the development of artificial feeding systems using membranes over heated blood for mass production of these vectors of disease [35]. Our results suggest that it is the change in RH accompanied by an increase in temperature that induces the biting response in flies coming from cooler and dryer conditions. We did not investigate the biting response of tsetse to RH increases at a constant temperature of 37°C for example or to temperature increases for flies maintained at high RH conditions. An increase in one factor, heat for example, may be sufficient to induce biting when a fly is already in a very humid environment or vice versa. Increasing temperature serves to increase the metabolic rate in tsetse [36] but, in the absence of an increase in humidity, a temperature increment alone is insufficient to induce the biting response. The water balance state of a fly could influence its eagerness to bite. Our flies were maintained under constant and high RH conditions before experiments. The role of water vapour can also differ according to the degree of haematophagy in a species: tsetse hardly responded to RH increments alone in this study whereas an increase only in humidity is sufficient to elicit probing in the stable fly that is both haematophagous and a nectar feeder [16,17] and in the nectar feeding hawkmoth [37].

As well as affecting response latency and the number of flies responding, increasing water vapour accompanied by an increase in temperature also increased the persistence of the biting response in *G. pallidipes*. This indicates that heat and humidity combined constitute an adequate stimulus to induce flies to persist in biting, a behaviour that represents a critical risk for tsetse [2]. Van Naters*, et al.*[14] found that two sensory modalities presented together, heat and uric acid, increased the biting persistence of *G. fuscipes fuscipes* compared to flies stimulated by heat alone. This interaction between sensory systems increased the time spent probing but not the numbers of bites. In our conditions the interaction between thermoreception and hygroreception increased the number of bites and correspondingly the time spent biting. Moreover, for a temperature increment of 13.1°C the average time per bite decreased with increasing RH above 34.4%. This suggests that the more adequate the stimulus, the quicker the fly withdraws its haustellum to try again when haustellum extension fails to reach the skin under optimum stimulus conditions, *i.e.* the haustellum is quickly brought back to initiate a new biting attempt. Since the average time between two consecutive bites remains unchanged, this suggests that flies repeat a stereotypic positioning behaviour between biting attempts that assists them to reach the skin through the host’s fur. The frequency of biting events was correlated with the amount of water in warm air but was not stable in time, being higher in the first 10s of the response, before falling rapidly thereafter. However, flies adapted more slowly at the higher dose of water vapour in warm air.

Neurones responded with a significant increase in spike frequency in all wpsw palp basiconic sensilla exposed to a decrease in RH. When these sensilla were also exposed to an increase of 9°C in dry air, no significant response in spike frequency was observed (data not shown). The overall responses represent complex spike patterns and our recordings did not permit to differentiate between the responses of the three neurones within the basiconic wpsw sensilla on the palps. However, we can conclude from our recordings that these sensilla contain one or more dry cells. Wet cells also occur in basiconic sensilla on the palps, but were very rare as only four were found in preliminary recordings from more than 60 sensilla. Tsetse are thus able to perceive RH changes with dry and wet cells increasing their spike firing frequency as humidity decreases or increases. To our knowledge, this is the first report of hygroreceptor cells in tsetse. Bursell [38] previously demonstrated that several tsetse species including *G. pallidipes* are more active in dry air than in moist air and that antennal removal did not modify the response, indicating that hygroreceptor cells responsible for the observed behaviour occur elsewhere on the fly. Evans and Gooding [18] showed that in the presence of CO_2_, moist and heated air is more efficient at eliciting upwind flight in tsetse than heated air alone. Temperature and humidity conditions affect water loss in tsetse flies [39], so hygroreceptor cells could evidently serve to guide flies in the selection of resting sites where they would be subject to lower rates of water loss, as proposed by Chown*, et al.*[40] and demonstrated in mosquitoes [41].

The ultra-structure of the basiconic wpsw sensilla housing the dry cells on the palps of *G. pallidipes* is very similar to typical olfactory wpsw sensilla of insects [42]. We recorded responses to the host-related volatiles 1-octen-3-ol, n-valeric acid and dimethyl-trisulphide from cells in these sensilla (data not shown), confirming earlier findings by Lewis [43] of responses by these cells to carboxylic acids. The fact that basiconic sensilla of insects can contain both hygro- and chemoreceptor cells and has already been documented (cf. [44]), but to our knowledge this has never been reported before for testse. All basiconic sensilla examined present 2 neurones with swollen dendrites that occupy all the lymphatic space at the base of each sensillum, separating the sensillar lymph into proximal and distal selections. The swelling remained unaffected by a hypertonic fixative solution. The transduction mechanism for the coding of differences in RH is unknown, but it appears clear that pressure exerted on the membrane of hygroreceptor cells could play a key role [45]. We propose that as RH levels drop, water loss from the wall-pore sensillum could cause the inflated dendrite to be deformed as a result of a pressure difference exerted on either side of the dendritic swelling.

## Conclusions

Our results show that temperature acts synergistically with water vapour increments in air in inducing the biting response in tsetse and that palp sensilla housing hygroreceptor cells are involved in the perception of differences in RH. As such, the tsetse biting response exploits the homoeothermic requirements of hosts where evaporative water loss is a crucial means of lowering body temperature. The findings also underline the perception of a substance as essential to life as water in the biting behaviour of obligate haematophagous insects like tsetse where host animals are the only source of both nutrients and imbibed water.

The experimental design described here that permits quantification of the biting response of tsetse to heat and humidity constitutes a means to study inhibition of this crucial response in trypanosome transmission. Disruption of either thermoreception or hygroreception is likely to be sufficient in considerably reducing the biting response. The experimental set up also provides a means to investigate the effect of trypanosome infection on tsetse biting responses.

## Competing interests

The authors declare no competing interest.

## Authors’ contributions

CCJF, BS and PMG designed the study; CCJF, BS and VM collected data; CCJF, BS, VM and PMG analysed data; CCJF, BS, VM and PMG wrote the paper. This study is a part of the Ph.D thesis of CCJF at the University of Neuchâtel. All authors read and approved the final version of the manuscript.

## Supplementary Material

Additional file 1 **Example of a video record used to quantify the biting responses of *G. pallidipes.*** The holding cage was slid from an acclimatisation airflow at 24.5°C, 7.3% RH to a test airflow (red light on) where the female was exposed to an increase of 13.1°C and 72.4% RH. The fly responded by regularly passing its haustellum through the cage netting. Latency is the time between the moment the red light turned on and the onset of the first bite, *i.e.* the first time the haustellum crosses the netting. To limit the size of the file only 22 s of the high resolution video record is shown. Click here for file

Additional file 2 **Time spent biting (A) and average time per bite (B) at increasing RH.** The temperature increment was 13.1°C. Boxplots with different letters are significantly different according to a post-hoc test following a GLM with a reciprocal link function (Gamma distribution) with a Tukey contrast matrix. For time spent biting at increments of 0.2% and 10.5% RH see legend to Figure 4. In B, one point is not shown (at 34.5%, 10.2 s) as the y-axis was limited to 4 s for purposes of readability. Click here for file

Additional file 3 **Neuronal responses in a *G. pallidipes *palp basiconic sensillum to RH decreases in the air.** Arrows indicate the onset of stimulation. Click here for file
