# Supplementary figures and images for: Water vapour and heat combine to elicit biting and biting persistence in tsetse
Source: Parasit Vectors. 2013 Aug 19;6:240. doi: 10.1186/1756-3305-6-240 (PMC3765185; doi:10.1186/1756-3305-6-240)

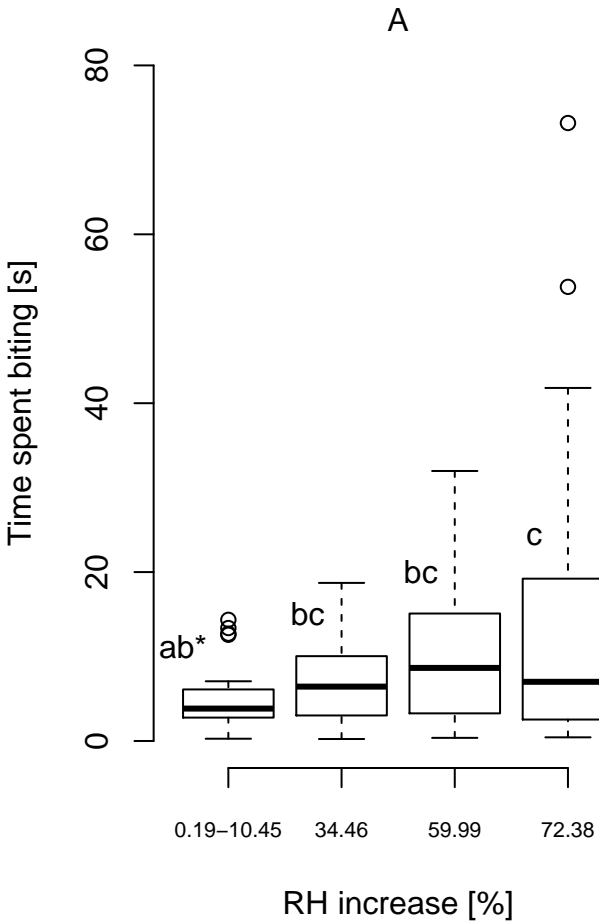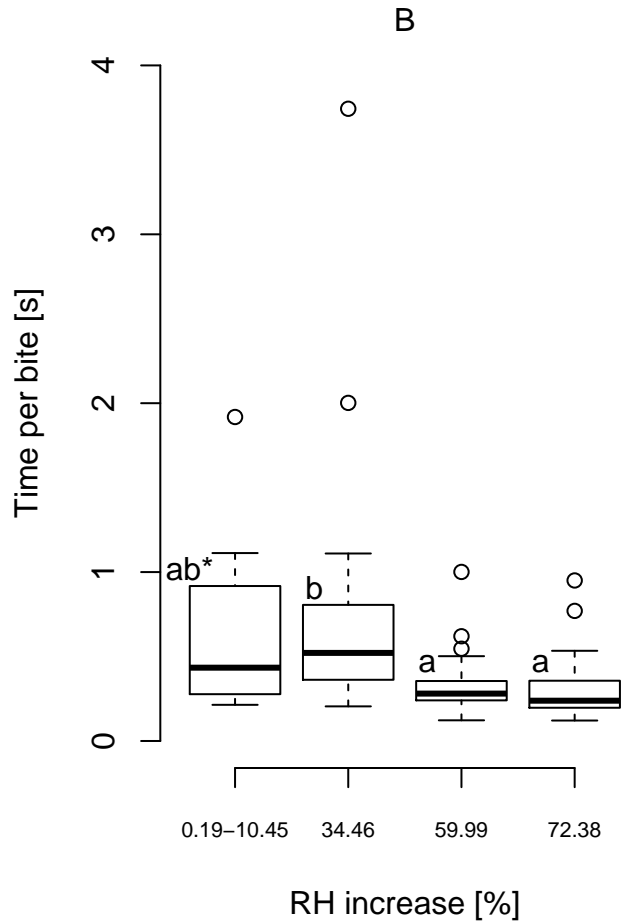

Supplement: Additional file 2 — Time spent biting (A) and average time per bite (B) at increasing RH. The temperature increment was 13.1°C. Boxplots with different letters are significantly different according to a post-hoc test following a GLM with a reciprocal link function (Gamma distribution) with a Tukey contrast matrix. For time spent biting at increments of 0.2% and 10.5% RH see legend to Figure 4. In B, one point is not shown (at 34.5%, 10.2 s) as the y-axis was limited to 4 s for purposes of readability. [file 1756-3305-6-240-S2.pdf]

$\Delta RH = 0.06$

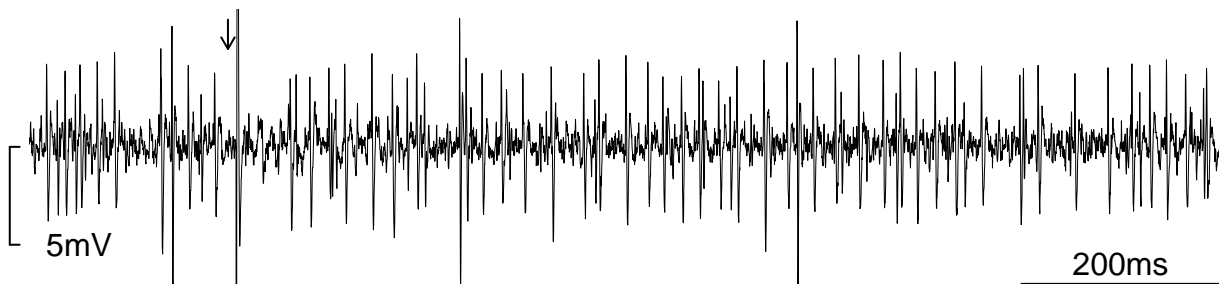

$\Delta RH = -27.35$

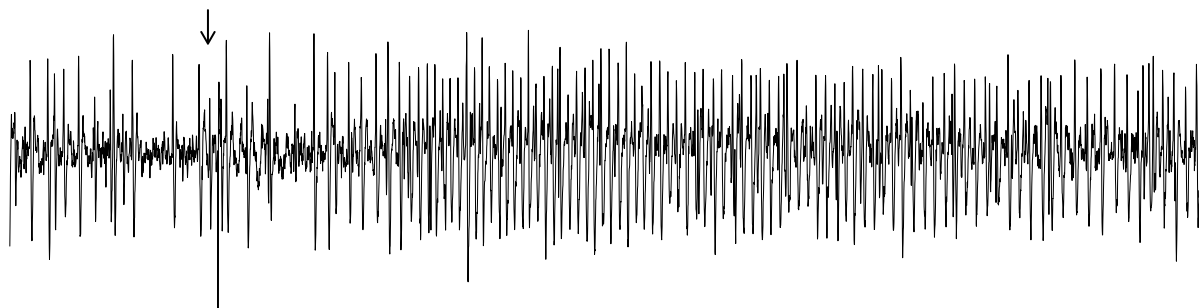

$\Delta RH = -41.44$

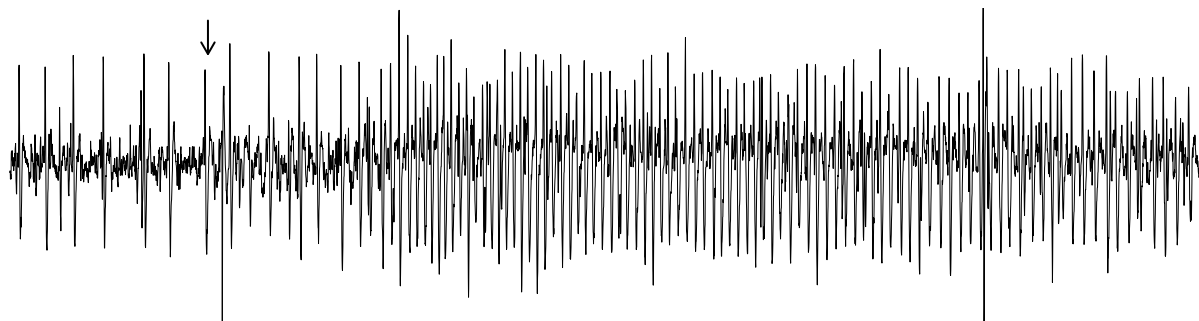

Supplement: Additional file 3 — Neuronal responses in a G. pallidipes palp basiconic sensillum to RH decreases in the air. Arrows indicate the onset of stimulation. [file 1756-3305-6-240-S3.pdf]
